# Illuminating the dynamics of signal integration in Natural Killer cells

**DOI:** 10.3389/fimmu.2012.00308

**Published:** 2012-10-04

**Authors:** Sophie V. Pageon, Dominika Rudnicka, Daniel M. Davis

**Affiliations:** Division of Cell and Molecular Biology, Imperial College LondonLondon, UK

**Keywords:** NK cells, immune synapse, signal integration, super-resolution imaging, microclusters, nanoclusters

## Abstract

Natural Killer (NK) cell responses are shaped by the integration of signals transduced from multiple activating and inhibitory receptors at their surface. Biochemical and genetic approaches have identified most of the key proteins involved in signal integration but a major challenge remains in understanding how the spatial and temporal dynamics of their interactions lead to NK cells responding appropriately when encountering ligands on target cells. Well over a decade of research using fluorescence microscopy has revealed much about the architecture of the NK cell immune synapse – the structured interface between NK cells and target cells – and how it varies when inhibition or activation is the outcome of signal integration. However, key questions – such as the proximity of individual activating and inhibitory receptors – have remained unanswered because the resolution of optical microscopy has been insufficient, being limited by diffraction. Recent developments in fluorescence microscopy have broken this limit, seeding new opportunities for studying the nanometer-scale organization of the NK cell immune synapse. Here, we discuss how these new technologies, super-resolution imaging and other novel light-based methods, can illuminate our understanding of NK cell biology.

## INTRODUCTION

Natural Killer (NK) cells are classified as lymphocytes of the innate immune system because they are capable of directly killing virus-infected or transformed cells without prior priming. Therefore their responses need to be tightly regulated to allow proper distinction between diseased cells and neighboring healthy tissues. To ensure this, NK cell activation is shaped by the balance of signals induced from inhibitory and activating germline-encoded receptors expressed on their surface. Integration of signals from cell surface receptors takes place across a dynamic structured interface formed at the contact between NK cells and target cells – the immune synapse (Davis et al., 1999). Assembly of the immune synapse occurs in sequential stages of receptor–ligand organization, cytoskeletal rearrangements, and cellular polarization, from initial adhesion to an appropriate response such as killing a target cell by the release of cytolytic granules (Orange et al., 2003).

NK cells are activated through surface receptors that can recognize signs of disease on other cells using different strategies. Receptors such as natural-killer group 2 member D (NKG2D) recognize ligands that are encoded in our genome and can be expressed or up-regulated on diseased or infected cells – a strategy sometimes called recognition of induced self. Alternatively, Fc receptor CD16 interacts with the Fc portion of antibodies to trigger an attack on opsonized target cells in the process of antibody-dependent cellular cytotoxicity (ADCC). Some other NK cell receptors, including natural cytotoxicity receptors NKp44 and NKp46, are less well understood. Engagement of activating receptors initiates downstream signaling cascades (reviewed in Vivier et al., 2004) that involve phosphorylation of Vav1 and can ultimately lead to the release of lytic granules or cytokines.

Killing of target cells can also be controlled by the ligation of inhibitory receptors, according to the “missing self” hypothesis (Kärre et al., 1986) - where loss of a protein normally found on cell surfaces can also serve as a signature of disease (Ljunggren and Kärre, 1990). In particular, healthy cells are protected by expression of class I major histocompatibility complex (MHC) proteins, which ligate inhibitory NK cell receptors, including the Killer-cell immunoglobulin-like receptors (KIR) family or the CD94/NKG2A complex. Inhibitory signals are initiated by phosphorylation of immunoreceptor tyrosine-based inhibition motifs (ITIMs) within the cytoplasmic region of KIR or CD94/NKG2A (Burshtyn et al., 1996). This leads to the recruitment of phosphatases SHP-1 and SHP-2, which initiate a signaling pathway that results in the dephosphorylation and hence inactivation of key components in the signaling pathway for activation, including Vav1 (Burshtyn et al., 1996; Stebbins et al., 2003). Many other inhibitory receptors are found on NK cells, such as TIGIT (Stanietsky et al., 2009) and siglecs (Crocker and Varki, 2001), but their roles currently remain somewhat enigmatic, and may be tissue-specific.

Despite the extensive literature on activating and inhibitory signals in NK cells, we do not clearly understand the sequence of events occurring while NK cells decide an appropriate response. This is broadly the gap in much of contemporary cell biology – we know which protein–protein interactions are important but have little understanding as to how all these interactions play out in space and time to give rise to complex cell behaviors, such as making a decision about the state of health of another cell. To understand how this works in detail requires knowing where and when protein–protein interactions occur to regulate NK cell behavior; and hence it is important to determine the spatio-temporal organization of the immune synapse at the appropriate – i.e. nanometer-scale. Here, we discuss recent advances in our understanding of spatial and temporal dynamics of signal integration in NK cells with an emphasis on how high-resolution imaging techniques have already contributed – and can contribute far more in the immediate future.

## MICROMETER-SCALE ORGANIZATION OF THE IMMUNE SYNAPSE

In the initial studies describing T cell and NK cell immune synapses, imaging at the micrometer-scale revealed that proteins at the interface between interacting cells segregated into distinguishable supramolecular clusters (Monks et al., 1998; Davis et al., 1999; Grakoui et al., 1999; Vyas et al., 2001). In the T cell synapse, the T cell receptor (TCR) was enriched in the central region, the central supramolecular activating cluster (cSMAC), where it colocalized with Lck and PKC signaling molecules, segregated from the adhesion molecule LFA-1 which formed a ring around the center named the peripheral SMAC (pSMAC). Similarly, in the cytolytic NK cell synapse, lytic granules accumulated at the cSMAC while adhesion molecules segregated into a pSMAC (Orange et al., 2003). These ‘prototypical’ types of organization are sometimes referred to as mature immune synapses.

The process of an immune cell meeting another cell and then moving on without effector functions being elicited should not necessarily be considered a non-event because, at least for NK cells, a structured interface does still assemble – a so-called inhibitory synapse – and specific signals trigger inhibition to allow the NK cell to move on (Davis et al., 1999). At the inhibitory synapse, proteins organize in such a way that KIR and associated components accumulate in central clusters segregated from LFA-1 at the periphery (Schleinitz et al., 2008). Intriguingly, segregation of KIR from integrins is determined by the density of interacting class I MHC protein on target cells (Almeida and Davis, 2006), indicating a link between the function of KIR – to assess levels of class I MHC expression – and its supramolecular organization.

The mechanisms by which proteins organize at the immune synapse are thought to involve the actin cytoskeleton, although whether by direct tethering to sub-synaptic actin filaments or confinement within an actin “picket-fence” meshwork remains unclear. It has also been proposed that proteins would segregate according to their localization into cholesterol-enriched lipid raft domains, in which activating receptors are concentrated and inhibitory receptors and phosphatases such as CD45 are excluded (Leupin et al., 2000). The polarization of lipid rafts to the immune synapse has been implicated in NK cell activation and has been shown to be blocked by the engagement of inhibitory receptors (Lou et al., 2000; Watzl and Long, 2003; Endt et al., 2007). Another potential mechanism is that proteins might spontaneously segregate within the synapse according to their size. Evidence for this, and its influence on signaling according to the “kinetic segregation” model, has come from both experimental data and mathematical modeling, both for T cells (Choudhuri et al., 2005; Davis and Van der Merwe, 2006; James and Vale, 2012) and NK cells (Köhler et al., 2010; Alakoskela et al., 2011; Burroughs et al., 2011). The precise scale over which proteins segregate by size remains, however, to be clarified.

## HIGH-RESOLUTION IMAGING OF IMMUNE SYNAPSES

One of the high-resolution techniques introduced early-on to visualize membrane-proximal events occurring at the immune synapse was total internal reflection fluorescence (TIRF) microscopy. This allows imaging of processes happening at the interface between a cell and a coverslip with strongly decreased background fluorescence (**Table 1**). Cells could be imaged when plated on activating surfaces, such as slides coated with antibodies or protein ligands, or protein-rich planar lipid bilayers. Particularly useful is that the protein composition of bilayers and coated slides can be precisely controlled – allowing quantitative studies regarding numbers of ligands used to stimulate immune cells. The application of TIRF microscopy to studies of immune synapses made it possible to observe proteins organize into discrete assemblies on a micrometer to sub-micrometer-scale, named microclusters, where (at least some) signaling takes place. Microclusters of TCR were detected and shown to be involved in signaling while migrating from the periphery of the synapse toward the center (Yokosuka and Saito, 2010) and this movement was dependent on cytoskeletal processes. Indeed, retrograde flow of actin occurring during synapse formation can carry microclusters of TCR in T cells and activating receptors in NK cells from the cell periphery toward the center (Varma et al., 2006; Kaizuka et al., 2007; Abeyweera et al., 2011). Interestingly, inhibitory NK cell receptors, KIR specifically, accumulate at the synapse largely independently of cytoskeletal processes (Davis et al., 1999; Standeven et al., 2004), likely moving to the synapse by passive diffusion and accumulating there by ligation within the synaptic cleft.

**Table 1:** High- and super-resolution techniques for imaging immune synapses at improved resolution.

Technique	Principle	Advantages	Limitations
Total internal reflection fluorescence (TIRF)	The excitation beam is directed onto the sample at a critical angle and is reflected off the coverslip-sample interface, generating an evanescent wave in which fluorophores are excited	- High sectioning strength (about 100 nm into the sample) - Minimal background noise from intracellular out-of-focus regions	- Limited to regions at or near the cell surface - Remains subject to the diffraction limit
Optical tweezers with confocal microscopy	Manipulation of live cells in all dimensions using a tightly focused laser beam that can trap particles; conjugates are oriented so that synapse is aligned to the imaging plane	- *En face* observation of the immune synapse at high speed and high resolution - Promotion of formation of specific cell conjugates	- Remains subject to the diffraction limit
Structured illumination microscopy (SIM)	Full-field illumination of the sample with spatially structured light generates images with high spatial frequency information that are reconstructed into a super-resolution image	- 3D resolution improvement - Possible to image deep within a cell - Conventional dyes can be used (if sufficiently photostable) - Straightforward sample preparation	- At best two-fold improvement in resolution - Complex post-acquisition processing of data (introduction of artefacts needs to be controlled) - Long acquisition and processing times
Stimulated emission depletion (STED) microscopy	The sample is scanned by two overlapping concentric laser beams to minimize the volume of detection: the first laser excites the fluorophores; the second laser of longer wavelength drives the fluorophores into the ground state by stimulated emission depletion	- Fast acquisition and potential for live cell imaging - Potential for extremely high resolution (5 nm) - Great depth penetration and potential for 3D imaging - No need for post-acquisition data processing (minimal artefacts)	- Problems with photobleaching in biological samples - Only some conventional dyes and fluorescent proteins can be used - Long acquisition time to collect sufficient photons
Stochastic optical reconstruction microscopy (STORM)	Individual fluorophores are stochastically excited, localized and bleached. A super-resolution image is reconstructed from individual localizations across thousands of frames	- Potential for very high resolution (10–20 nm) and single-molecule data - 3D resolution improvement possible when combined with TIRF or a cylindrical lens - Conventional dyes can be used	- Long acquisition times - Complex and time-consuming post-acquisition image analysis - Possibility of artefacts due to free dye or free labeled antibody - Limited to fixed cells
Photoactivated localization microscopy (PALM)	Principle is similar to STORM, but relies on genetically encoded photoswitchable fluorescent proteins	- Potential for very high resolution (10–20 nm) and single-molecule data - 3D resolution improvement possible when combined with TIRF or a cylindrical lens - Compatible with live cell imaging	- Long acquisition times - Complex and time-consuming post-acquisition image analysis - Lower photon counts from fluorescent proteins might lead to poorer resolution improvement
Fluorescence correlation spectroscopy (FCS)	Correlation analysis of fluctuations in fluorescence intensity within a small confocal volume reveals information about diffusion, concentration, and dynamics of molecules	- Conventional dyes and fluorescent proteins can be used - Used with confocal or multi-photon microscopy - Single-molecule sensitivity	- Indirect measurements - Complex curve fitting - Long acquisition times

Inhibitory NK cell KIR proteins also organize into microclusters (Oddos et al., 2008; Abeyweera et al., 2011). Phosphorylation of KIR2DL1 was visualized using fluorescence lifetime imaging (FLIM) to detect Förster resonance energy transfer (FRET; Treanor et al., 2006). This enabled detection of discrete signaling microclusters of KIR2DL1 at the synapse and provided the first indication that NK cell signaling may occur within small protein assemblies. Subsequently, the use of optical tweezers with confocal microscopy to study T and NK cell synapses revealed the presence of micron-scale clusters of protein at the contact between two cells rather than at the interface between one cell and a coverslip (Oddos et al., 2008). An increased resolution was achieved by avoiding the need for an *en face* 3D reconstruction from multiple planes imaged by confocal microscopy by reorienting conjugates so that the immune synapse lies horizontally in the imaging plane (**Table 1**). This study established that microclusters of KIR2DL1 within the inhibitory NK cell synapse moved from the synapse periphery to the center (Oddos et al., 2008).

At the lytic synapse activating receptor NKG2D also accumulated in microclusters at the cell periphery that then moved centripetally (Abeyweera et al., 2011), forming a ring around a central secretory domain through which release of lytic granules occurred (Liu et al., 2009; Brown et al., 2011). Similarly, microclusters of CD16, as visualized through their interaction with fluorescent Fc portions of antibodies in supported lipid bilayers, formed at the periphery of the contact and moved toward the center of the synapse (Liu et al., 2012). Assembly of these microclusters was impaired when inhibitory receptor CD94/NKG2A was co-engaged, indicating their formation was already an outcome of signal integration. Inhibition was partially mediated by phosphorylation of Crk, a small adaptor protein that with other proteins forms a cytoskeleton scaffold complex, which regulates actin rearrangements (Peterson and Long, 2008). Upon phosphorylation, Crk dissociated from the scaffold complex, disrupting F-actin at the center of the synapse, thereby preventing movement of CD16 microclusters and transduction of activating signals (Liu et al., 2012). Interestingly, engagement of CD94/NKG2A in the absence of co-engagement of other receptors was sufficient to induce Crk phosphorylation. This suggests a dual function for ITIM-bearing receptors, whereby inhibitory signaling can be induced even in the absence of activation in order to remove physical constraints on activating receptors and “license” cells for subsequent engagement of activating receptors (Liu et al., 2012).

## USING PHOTOCHEMISTRY TO PROBE THE IMMUNE SYNAPSE

Peptides can be made to include a photocleavable group protecting a functionally important site for recognition that can be cleaved by light to yield the native active version, thus enabling the time-resolved study of fast biological processes. DeMond et al. (2006) exploited a caged antigenic peptide presented by class II MHC protein to selectively activate CD4^+^ T cells on a supported lipid bilayer in a spatially and temporally controlled way, demonstrating the strategy for examining the arrangements of the T cell immune synapse. Independently, Huse and colleagues developed a photocleavable peptide to study the temporal and spatial aspects of T cell signaling and protein organization at the synapse (Huse et al., 2007; Quann et al., 2009, 2011).

Huse and colleagues also extended this approach to study NK cells (Abeyweera et al., 2011). It had been established previously that large residues in the P8 position of peptides in HLA-C can mask the binding site for KIR (Rajagopalan and Long, 1997; Boyington et al., 2000). By introducing a large photocleavable moiety on this residue, peptide–MHC complexes are not recognized by KIR until irradiation with UV light. Such a photolabile peptide, in complex with HLA-Cw3, was introduced into lipid bilayers containing activating ligand ULBP3 and integrin ICAM-1, and used to precisely control the timing of inhibition via KIR2DL2 (Abeyweera et al., 2011). Inhibition was induced at various time points after NK cells contacted the bilayer, to study how early inhibitory signaling needs to be initiated to impair the process of activation. Interestingly, the authors observed that initiation of inhibition up to 15 min after the NK cells landed was still able to reverse activation. These results suggest that a cell is never fully committed to killing and is continuously integrating signals induced by surface receptors. This is consistent with earlier imaging using slides coated with micro-patterned stripes of activating and inhibitory ligands, which also demonstrated that a continued dominance of local activating signals is necessary for a stimulatory NK cell response (Culley et al., 2009).

## NANOMETER-SCALE ORGANIZATION OF THE IMMUNE SYNAPSE REVEALED BY SUPER-RESOLUTION MICROSCOPY

Many different super-resolution techniques capable of nanometer-scale resolution have now been demonstrated. The principles behind some of these techniques along with their main advantages and limitations are summarized in **Table 1**. Although their application to immune cell biology is very new, they have already led to some important discoveries about the nanometer-scale organization of proteins at immune synapses. Super-resolution images obtained using structured illumination microscopy (SIM) revealed how the cortical actin mesh – which underlies all cell surface membranes – is remodeled upon NK cell activation (Brown et al., 2011). Observing actin at the synapse with such resolution revealed that the periodicity of the actin mesh is increased to create holes sized to fit lytic granules. Independently, and published at the same time, another super-resolution microscopy technique stimulated emission depletion (STED) microscopy also revealed an opening of the actin mesh in domains within the synapse center (Rak et al., 2011). Together, these findings refute the earlier dogma that actin was entirely cleared from the center of the sub-synaptic area in order to allow release of lytic granules.

Super-resolution imaging can examine far more than just actin and immunologists have begun to exploit its potential for imaging the nanometer-scale organization of receptors and ligands at synapses – with most studies so far focused on T cell synapses. Photoactivated localization microscopy (PALM) has been applied by several groups (Lillemeier et al., 2010; Owen et al., 2010; Sherman et al., 2011; Williamson et al., 2011), focused on visualizing the distribution and interaction of TCR and membrane-proximal signaling proteins and adaptors, such as LAT, during T cell activation. One study found TCR and LAT to be pre-clustered in quiescent T cells (Lillemeier et al., 2010). Upon activation of T cells, these small aggregates – or protein islands as the authors termed them – concatenated but surprisingly, instead of overlapping, TCR and LAT remained in distinct domains that became juxtaposed to each other. In a subsequent study using PALM, Sherman et al. (2011) also observed LAT in pre-existing nanoclusters in resting T cells. The authors suggest that by using PALM, what was previously detected as microclusters can now be visualized as a group of smaller subunits – nanoclusters, containing only few molecules. Two-color PALM extended these findings to demonstrate that TCR and ZAP-70 nanoclusters colocalized after TCR ligation. However, clusters of TCR were largely segregated from LAT clusters, with sparse regions of overlap potentially representing “hot spots” where LAT phosphorylation is triggered by ZAP-70 (Sherman et al., 2011). These findings highlight the importance of super-resolution imaging to disentangle contradictory models for immune receptor signaling dynamics. With the knowledge we now have, it is clear that proteins known to interact often transiently meet via the movements of nanometer-scale protein clusters to facilitate signaling – a process somewhat distinct from the textbook-level version of events involving a linear cascade of single protein–protein interactions.

Using TIRF microscopy as well as optical tweezers coupled with confocal microscopy, motile sub-synaptic vesicles containing LAT were seen to repeatedly move between clusters of SLP-76, where they decreased motility, highlighting that both vesicular LAT and surface clusters of LAT can play a role in TCR signaling (Purbhoo et al., 2010). A subsequent study combining PALM, live PALM, and stochastic optical reconstruction microscopy (STORM) indicated that TCR signaling may depend only on LAT molecules associated with sub-synaptic intracellular vesicles rather than LAT clusters already present in the plasma membrane (Williamson et al., 2011). Together, these two studies have led to the proposal of a new model in which vesicular traffic to and from the membrane is important for the recruitment and disassembly of signaling complexes – another refinement to the textbook-level description of events following TCR ligation.

Clearly, much can be gained by the application of super-resolution imaging to the study of NK cell signaling – to detect small changes in protein localization, nanocluster size distribution and molecular density within clusters during the process of target cell recognition. Indeed, the importance of the nanoscale organization of receptors was highlighted in a recent study using fluorescence correlation spectroscopy (FCS) using murine NK cell models (Guia et al., 2011). By recording the transit time of cell surface proteins moving through differently sized volumes illuminated by laser light and comparing to freely diffusing molecules, Guia et al. (2011) presented striking evidence that NK cell receptors were differentially restricted in their movement at the cell surface. Specifically, the authors proposed that activating receptors were compartmentalized by the organisation of the plasma membrane, while inhibitory receptors were confined by the actin meshwork. Surprisingly, at the surface of hyporesponsive, i.e. incompetent, NK cells, activating and inhibitory receptors were confined together by the actin meshwork. Thus, the authors suggested, altering the membrane organization of activating receptors may be important to how NK cell tolerance is achieved (Guia et al., 2011).

## CONCLUDING REMARKS

By applying techniques drawn from chemistry and physics, it becomes possible to interrogate important problems in biology – such as deciphering spatial and temporal details of signal integration in NK cells. High and super-resolution imaging has already established the importance of the nanometer-scale organization of proteins in T and NK cell membranes for appropriate signaling outcomes. An important paradigm emerging from the studies described here is that interactions between receptors and signaling proteins are controlled in part by the dynamics of supramolecular clusters rather than by isolated protein–protein interactions as once imagined. In the near future, super-resolution imaging techniques will reveal much about the spatial and temporal dynamics that underlie NK cells making appropriate decisions during immune surveillance of target cells.

## Conflict of Interest Statement

The authors declare that the research was conducted in the absence of any commercial or financial relationships that could be construed as a potential conflict of interest.
